# Effect of sample time on urinary lithogenic risk indexes in healthy and stone-forming adults and children

**DOI:** 10.1186/s12894-018-0430-8

**Published:** 2018-12-19

**Authors:** Adrian Rodriguez, Concepcion Saez-Torres, Concepcion Mir, Paula Casasayas, Nuria Rodriguez, Dolores Rodrigo, Guiem Frontera, Juan Manuel Buades, Cristina Gomez, Antonia Costa-Bauza, Felix Grases

**Affiliations:** 1 Laboratory of Renal Lithiasis Research, University Institute of Health Sciences Research (IUNICS-IdISBa), University of Balearic Islands, Ctra Valldemossa, km 7.5, 07122 Palma de Mallorca, Spain; 2 Department of Pediatric Nephrology, Son Espases Universitary Hospital, 07020 Palma de Mallorca, Spain; 3 Department of Urology, Son Llatzer Hospital, 07198 Palma de Mallorca, Spain; 4 Research Unit, Son Espases Universitary Hospital, 07020 Palma de Mallorca, Spain; 5 Department of Nephrology, Son Llatzer Hospital, 07198 Palma de Mallorca, Spain; 6 Clinical Analysis Service, Son Espases Universitary Hospital, 07020 Palma de Mallorca, Spain

**Keywords:** 12-h night urine, AP(CaOx) index, AP(CaP) index, Ca/Cit ratio, Crystallization risk, Renal Lithiasis

## Abstract

**Background:**

The diagnosis and follow-up of stone forming patients is usually performed by analysis of 24-h urine samples. However, crystallization risk varies throughout the day, being higher at night. The main objective of this study is to evaluate the urinary crystallization risk in adults and children by calculating risk indexes based on different collection periods.

**Methods:**

The study included 149 adults (82 healthy and 67 stone-formers) and 108 children (87 healthy and 21 stone-formers). 24-h urine was collected, divided into 12-h daytime sample (8 am to 8 pm), and 12-h overnight sample (8 pm to 8 am next morning). Solute concentrations, the calcium to citrate ratio (Ca/Cit), and the ion activity product of calcium oxalate (AP[CaOx]) and calcium phosphate (AP[CaP]) were calculated in each 12-h sample and in overall 24-h urine. Assessments were also related to stone type.

**Results:**

Ca/Cit and AP(CaOx) were significantly higher in stone forming patients than in healthy subjects. The 12-h overnight samples had the highest values for both risk indexes, confirming a greater risk for crystallization at night. The AP(CaP) index was significantly higher in patients with pure hydroxyapatite stones than healthy controls, but was not significantly different between stone-formers overall and healthy controls.

**Conclusions:**

The calculation of risk indexes is a simple method that clinicians can use to estimate crystallization risk. For this purpose, the use of 12-h overnight urine may be a reliable alternative to 24-h collections.

## Background

Renal lithiasis has a high prevalence in adults and is relatively rare in children, although it is increasing in all age groups [1, 2]. Up to 80% of renal stones contain different forms of calcium oxalate or calcium phosphate crystals [3, 4]. The different types of renal stones have different etiologies, which include particular features of urine composition. Thus, renal calculi consisting of calcium oxalate dihydrate (COD), pure hydroxyapatite (HAP), or admixtured COD+HAP, are more likely to form in the presence of urine with a high level of calcium and a low level of citrate [5]. On the other hand, even at relatively low levels of urinary supersaturation, injured papillary tissue can initiate the formation of papillary calcium oxalate monohydrate calculi (COMp) [6], and the presence of heterogeneous nucleating elements is related to the formation of unattached calcium oxalate monohydrate calculi (COMu) [5].

The traditional diagnosis and follow-up of stone-forming patients consists of assessment of the concentration and excretion of different solutes in 24-h urine samples (typically creatinine, calcium, magnesium, phosphate, oxalate, citrate, and uric acid), and measurements of urinary pH and volume. This approach allows diagnosis of metabolic abnormalities, such as hypercalciuria, hyperoxaluria, and hypocitraturia. However, some patients are classified as having idiopathic renal stones if there is no evidence of a metabolic abnormality. This may be because the key factor for urinary crystallization is not the absolute solute excretions but the urinary supersaturation degree, a parameter that depends on urine concentration. For this reason, and due to the multifactorial characteristics of renal lithiasis, some authors have developed different risk indexes to estimate the risk of urinary crystallization [7, 8]. Several studies used these indexes, including the calcium/citrate ratio, the ion activity product of calcium oxalate (AP[CaOx]), and calcium phosphate (AP[CaP]), to compare stone-forming children and adults with healthy controls, and found significant differences [7, 9–11]. Furthermore, previous studies have shown cut off values for urinary solute concentrations that makes urine prone to crystallize in an in vitro model [12, 13].

On the other hand, when performing the metabolic evaluation of the stone forming patient, it must be considered that urinary composition varies throughout the day, leading to a higher risk of crystallization at night. More precisely, there is a 12-h high-risk period from 8 p.m. to 8 a.m. [14]. However, to our knowledge, no study has yet compared the stone risk factors in 12-h overnight samples with 12-h daytime urine samples.

Thus, the objectives of this study are to *(i)* evaluate the urinary crystallization risk by measuring urinary solute concentrations and calculating risk formulas in children and adults with and without a history of lithiasis, *(ii)* compare the results of the risk parameters in 12-h daytime, 12-h overnight, and overall 24-h urine, and *(iii)* examine the relationships of the different risk formulas with stone type.

## Methods

### Study subjects

This study examined 257 participants who were divided into four groups: 87 healthy children (4–17 years), 21 stone-forming children (4–17 years), 82 healthy adults (23–57 years), and 67 stone-forming adults (18–71 years). Healthy children were recruited from schools, both primary and secondary. Stone formers were from the pediatric nephrology unit or the urology department of our tertiary hospital. All stone-formers had a confirmed history of renal lithiasis in the previous 2 years. Subjects with a history of disorders that could affect urine chemistry (bowel disease with malabsorption, bone fracture, active urinary tract infection, chronic kidney disease, metabolic syndrome) were excluded. Medications, including diuretics and alkali citrate, were discontinued three days before urine collection. Participants were told not to change their normal diet and physical activity. We obtained approval from the local Ethics Committee (IB3152/16) and informed consent from each participant or his/her legal representative.

### Renal calculi analysis

Stone analysis was performed by stereoscopic microscopy (Optomic, Madrid, Spain), scanning electron microscopy (S-530 M, Hitachi, Tokyo, Japan), X-ray microanalysis (Oxford Link Isis; Oxford, UK), and infrared spectrometry (Infrared Spectroscope Bruker IFS66; Bruker, Ettlingen, Germany).

The obtained stones were classified into 5 groups [5]: calcium oxalate monohydrate renal calculi developed on papillary tissue (COMp); unattached calcium oxalate monohydrate calculi (COMu); calcium oxalate dihydrate calculi (COD); calcium oxalate dihydrate-hydroxyapatite mixed calculi (COD+HAP); and hydroxyapatite (HAP) calculi. In recurrent stone-formers with different types of calculi, the last calculus was used for classification.

### Urine collection and analysis

Twenty-four-hour urine was collected in two separate flasks with thymol. The 12-h daytime sample began at 8 a.m. (after discarding first morning urine) and ended at 8 p.m.; at this time, participants were instructed to perform a micturition in the daytime bottle. The 12-h nighttime sample began at 8 p.m. and was collected until 8 a.m. on the next day (fasting state). Sampling adequacy was determined by asking participants about the completeness of urine collection and by using the recently-reported anthropometry-based age and sex-specific reference values for 24-h urinary creatinine excretion [15, 16].

Urinary volume, pH (measured using a Crison pH-meter), and the concentrations of creatinine, calcium, phosphorus, oxalate, uric acid, citrate, and magnesium were determined. Phosphorus was measured by the ammonium molybdate reduction method, magnesium by an enzymatic assay, calcium by a colorimetric reaction with Arsenazo III calcium-sensitive dye, uric acid by the uricase method, and creatinine using the Jaffe method. These analyses were performed using an Architect C16000 Autoanalyzer (Abbott Diagnostics, Illinois, USA). Urinary citrate was measured by an enzymatic assay (Biosystems, Barcelona, Spain), and urinary oxalate was determined using the oxalate oxidase/peroxidase method (LTA, Milano, Italy). All parameters were measured separately in 12-h samples, and then calculated for the overall 24-h urine.

The crystallization risk of urine was determined by the calcium-to-citrate ratio (Ca/Cit) and two modified estimates of AP(CaOx) and the AP(CaP), as described by Tiselius [17, 18]:

1 $$ \mathrm{AP}\left(\mathrm{CaOx}\right)\ \mathrm{index}=\mathrm{A}\times {\mathrm{Ca}}^{0.84}\times \mathrm{Ox}\times {\mathrm{Mg}}^{-0.12}\times {\mathrm{Cit}}^{-0.22}\times {\mathrm{V}}^{-1.03} $$

in which A is 2.7 for a 12-h sample, and 1.9 for a 24-h sample.

2 $$ \mathrm{AP}\left(\mathrm{CaP}\right)\ \mathrm{index}=0.0032\times {\mathrm{Ca}}^{1.07}\times {\mathrm{P}}^{0.70}\times {\left(\mathrm{pH}-4.5\right)}^{6.8}\times {\mathrm{Mg}}^{-0.12}\times {\mathrm{Cit}}^{-0.20}\times {\mathrm{V}}^{-1.31} $$

which was only calculated for 12-h samples, because pH was not determined for 24-h samples.

### Statistical analysis

Descriptive data are presented as medians and interquartile ranges. The Wilcoxon sum-rank test was used to compare groups. The Wilcoxon signed-rank test was used to compare daytime and nighttime samples. After examined the Bonferroni, Holm and Hochberg corrections, we considered a *p*-value of 0.001 or less as statistically significant. IBM SPSS Statistics version 22 ® for Windows was used for statistical analyses.

## Results

There were 257 study participants. The healthy and stone-forming adults had similar anthropometric characteristics, as did the healthy and stone-forming children (Table 1). Among adult stone formers, 11 had COMp stones, 11 had COMu stones, 18 had COD stones, 10 had HAP + COD stones, 4 had HAP stones, and stone analysis was unavailable for 13 patients. Among stone-forming children, 9 had COD stones, 1 had HAP + COD stones, and stone analysis was unavailable for the other 11 children.

**Table 1:** Anthropometric measures of the four study groups

	Healthy adults (*N* = 82)	Stone forming adults (*N* = 67)	Healthy children (*N* = 87)	Stone forming children (*N* = 21)
% men	46	55	57	64
Age (years)	40 (10)	46 (13)	12 (3)	12 (4)
Weight (kg)	68 (13)	71 (16)	46 (14)	44 (18)
Height (cm)	170 (8)	167 (10)	151 (17)	147 (21)
BMI (kg/m^2^)	24 (3)	25 (4)	19 (3)	19 (9)

Results are expressed as % or mean (SD)

Table 2 summarizes the urinary volume and solute concentrations in 24-h urine samples. Oxalate concentration was significantly higher in stone-forming than healthy adults; calcium concentration was significantly higher in stone-forming than healthy children.

**Table 2:** Urinary volume and solute concentrations in 24-h urine samples of the four study groups

	Adults			Children		
	Healthy	Stone formers	*p*-value	Heatlhy	Stone formers	*p*-value
Volume (mL/24 h)	1573 (1189–2173)	1778 (1250–2380)	0.089	876 (672–1180)	1100 (645–1707)	0.111
Creatinine (mg/L)	919 (648–1230)	804 (571–1098)	0.106	970 (763–1294)	796 (565–1245)	0.007
Calcium (mg/L)	106 (70–179)	124 (89–174)	0.166	72 (45–120)	130 (72–231)	< 0.001
Magnesium (mg/L)	64 (44–86)	55 (43–76)	0.143	113 (77–142)	93 (52–125)	0.081
Oxalate (mg/L)	15 (12–19)	20 (14–24)	0.001	22 (17–29)	25 (22–32)	0.120
Phosphorous (mg/L)	517 (397–784)	515 (357–637)	0.207	840 (597–1063)	597 (430–768)	0.002
Uric acid (mg/L)	369 (272–513)	343 (258–481)	0.334	548 (393–691)	411 (298–606)	0.018
Citrate (mg/L)	435 (286–676)	322 (226–470)	0.003	517 (373–722)	347 (205–626)	0.005

Results are expressed as median (P_25_-P_75_). Statistical comparisons are between healthy and stone-forming adults, and between healthy and stone-forming children

**Fig. 1:**
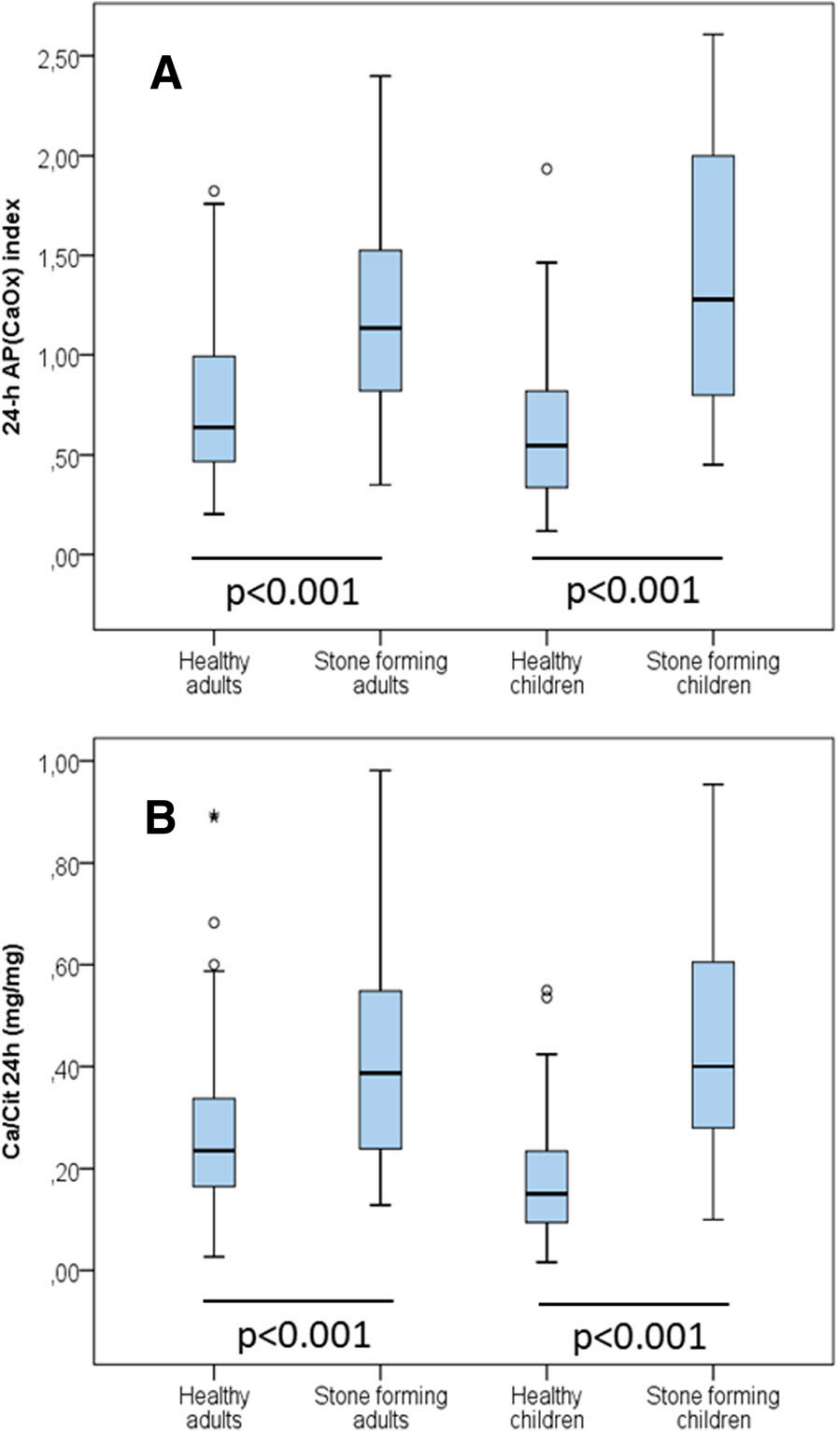
AP(CaOx) index (**a**) and Ca/Cit ratio (**b**) in 24-h urine samples of the four study groups. Statistical comparisons are between healthy and stone-forming adults, and between healthy and stone-forming children

The AP(CaOx) index and Ca/Cit ratio in 24-h urine samples were significantly higher in stone-formers than in healthy subjects, among children and adults (*p* < 0.001 for both comparisons) (Fig. 1).

Measurements of risk indexes in 12-h samples are shown in Table 3 (adults) and Table 4 (children). Regarding AP(CaOx) index and Ca/Cit, significant differences between patients and healthy subjects were observed when we performed the comparisons using only the 12-h day sample or the 12-h night urine fractions (*p* < 0.001). On the contrary, for the AP(CaP) index in 12-h samples, the differences did not reach statistical significance, neither in the daytime nor in the overnight sample analysis.

**Table 3:** Urinary volume, pH, solute concentrations, Ca/Cit ratio, and AP indexes in 12-h daytime and 12-h overnight urine samples of healthy and stone-forming adults

	Healthy adults (n = 82)			Stone forming adults (n = 67)		
	12-h day	12-h night	*p*-value	12-h day	12-h night	*p*-value
Volume (mL/12 h)	870 (600–1165)	650 (481–1010)	0.001	920 (700–1200)	780 (600–1135)	0.071
Creatinine (mg/dL)	940 (560–1310)	1025 (670–1563)	0.002	736 (562–1233)	946 (553–1224)	0.192
Calcium (mg/L)	90 (72–161)	133 (63–201)	0.060	123 (84–167)	142 (90–184)	0.031
Magnesium (mg/L)	52 (37–81)	79 (50–109)	< 0.001	49 (38–68)	68 (50–90)	< 0.001
Oxalate (mg/L)	15 (11–19)	16 (11–23)	0.04	18 (14–24)	21 (15–27)	0.003
Phosphorous (mg/L)	469 (353–683)	651 (442–1085)	< 0.001	473 (314–622)	581 (382–773)	< 0.001
Uric acid (mg/L)	426 (278–556)	372 (226–526)	0.259	395 (263–499)	338 (241–473)	0.031
Citrate (mg/L)	495 (315–754)	408 (232–710)	0.016	347 (223–531)	324 (205–434)	0.047
pH	6.27 (5.95–6.71)	5.64 (5.43–5.98)	< 0.001	6.12 (5.71–6.60)	5.79 (5.49–6.16)	< 0.001
Ca/Cit (mg/mg)	0.21 (0.15–0.30)	0.25 (0.18–0.42)	< 0.001	0.34 (0.21–0.44)	0.43 (0.26–0.64)	< 0.001
AP (CaOx) index	0.64 (0.49–0.90)	0.66 (0.46–1.13)	0.057	1.10 (0.66–1.54)	1.35 (0.90–1.84)	0.001
AP (CaP) index	4.85 (1.09–18.24)	0.33 (0.06–2.49)	< 0.001	3.41 (0.36–15.30)	0.93 (0.07–7.87)	0.001

Results are expressed as median (P_25_-P_75_)

**Table 4:** Urinary volume, pH, solute concentrations, Ca/Cit ratio, and AP indexes in 12-h daytime and 12-h overnight urine samples of healthy and stone-forming children

	Healthy children (n = 87)			Stone forming children (n = 21)		
	12 h day	12 h night	*p*-value	12 h day	12 h night	*p*-value
Volume (mL/12 h)	480 (378–708)	360 (300–450)	< 0.001	650 (335–930)	530 (315–705)	0.192
Creatinine (mg/dL)	908 (686–1250)	1162 (835–1469)	< 0.001	886 (507–1393)	1000 (611–1097)	0.274
Calcium (mg/L)	57 (38–99)	86 (41–167)	< 0.001	181 (57–209)	150 (101–258)	0.082
Magnesium (mg/L)	77 (54–110)	151 (110–198)	< 0.001	79 (51–100)	128 (55–163)	0.021
Oxalate (mg/L)	20 (15–27)	26 (19–34)	< 0.001	24 (20–32)	27 (23–37)	0.244
Phosphorous (mg/L)	630 (430–872)	1060 (804–1414)	< 0.001	450 (337–664)	870 (480–1094)	0.001
Uric acid (mg/L)	597 (431–720)	529 (373–632)	0.002	454 (266–646)	396 (271–578)	0.244
Citrate (mg/L)	557 (405–737)	462 (328–668)	< 0.001	379 (250–710)	299 (208–477)	0.004
pH	6.60 (6.15–6.96)	5.79 (5.60–6.22)	< 0.001	6.36 (6.08–7.12)	5.91 (5.55–6.23)	0.001
Ca/Cit (mg/mg)	0.11 (0.07–0.17)	0.21 (0.10–0.36)	< 0.001	0.40 (0.21–0.54)	0.50 (0.33–0.94)	0.002
AP (CaOx) index	0.46 (0.29–0.65)	0.67 (0.34–1.13)	< 0.001	1.12 (0.74–1.80)	1.28 (0.87–2.55)	0.052
AP (CaP) index	8.24 (2.34–21.86)	1.09 (0.16–3.04)	< 0.001	8.85 (1.78–62.5)	3.03 (0.34–5.56)	0.014

Results are expressed as median (P_25_-P_75_)

Tables 3 and 4 also compare the urinary parameters of daytime and overnight samples. Healthy and stone-forming adults had significant differences in magnesium, phosphate, urinary pH, Ca/Cit, and AP(CaP). On the contrary, healthy subjects had a lower nighttime urinary volume, and stone-formers had a greater nighttime AP(CaOx). Comparison of daytime and nighttime samples in children indicated the healthy children had significant differences in all parameters except uric acid concentration. On the contrary, in the stone forming children group, some differences did not reach significance due to the small size of the sample.

**Fig. 2:**
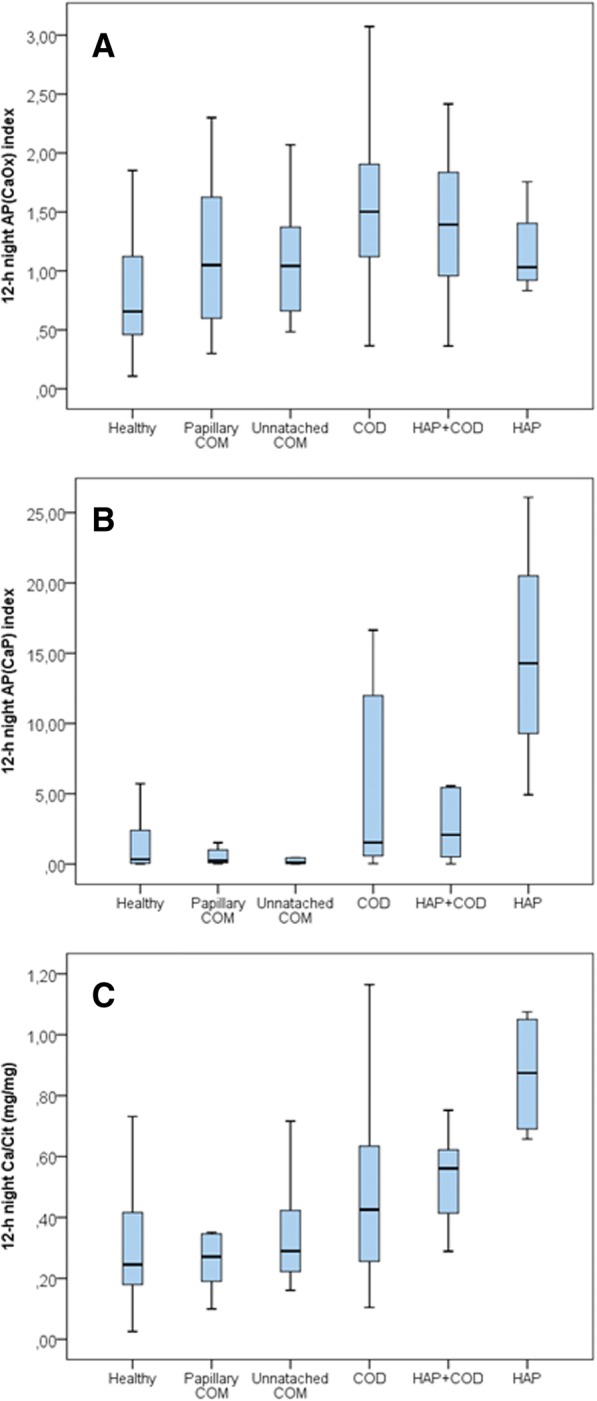
AP(CaOx) index (**a**), AP(CaP) index (**b**), and Ca/Cit ratio (**c**) in 12-h overnight urine samples of adult stone-formers according to calculus composition

Figure 2 shows the AP(CaOx) index, AP(CaP) index, and the Ca/Cit ratio for the 12-h overnight urine samples of stone-forming adults according to calculus composition. Patients whose calculi were COD or COD + HAP had higher AP(CaOx) indexes, while patients with pure HAP calculi had higher AP(CaP) indexes. Patients with COD, HAP, and COD+HAP calculi had higher Ca/Cit ratios. The evaluation of risk indexes in relation to stone composition performed in 12-h daytime urine and in 24-h urine showed a similar pattern of differences than observed in the 12-h overnight urine, although for AP(CaP) there were more overlapping results in the daytime than in overnight samples (data not shown).

## Discussion

The main findings of this study are that the AP(CaOx) and Ca/Cit values were significantly higher in the urine of stone-forming children and adults than in the corresponding healthy controls, being the results also evident by the only analysis of the 12-h overnight urine fraction. These indexes vary according to stone composition in adults. In addition, the AP(CaP) index was only elevated in adults with phosphate stones.

Previous studies have repeatedly stressed the important role of urine supersaturation in the genesis of renal calculi [8]. However, neither the European Association of Urology nor the American Urological Association includes calculation of supersaturation in their guidelines for evaluation of patient with renal lithiasis [19, 20]. In fact, some authors do not perform these measurements because they believe there is only limited evidence that monitoring of supersaturation in urine can prevent stone recurrence [21]. On the contrary, other authors have stated that assessment of urinary lithogenic risk, determined by measuring data related to the extent of supersaturation, is useful for guiding treatment and checking patient compliance [22, 23]. The use of specific easy-calculating formulas for estimation of supersaturation overcomes several methodological pitfalls of other procedures [7, 8, 10]. We found that AP(CaOx) and Ca/Cit values provided reliable estimates of the risk of renal lithiasis, in that each was significantly higher in stone-forming children and adults than the corresponding healthy controls. In the case of the AP(CaP) index, we observed differences in patients with phosphate stones, but not stone-formers overall, because most subjects had calcium oxalate stones.

Use of the Ca/Cit ratio to assess the risk of renal lithiasis is widespread in the literature and in clinical practice [9]. Calculation of AP indexes allows integration of information on additional components of urine, as well as urine volume. Regarding cut-off points for the different indexes, the overlap of index values in the healthy and stone-forming groups means it is difficult to establish precise values to discriminate subjects with and without risk. Despite this, we believe these indexes provide relevant information, because higher index values indicate higher risk of crystallization in urine [24].

Some researchers have recommended that supersaturation be assessed in 24-h urine samples before treatment, 4–6 weeks afterwards, and in subsequent follow-ups [25]. However, collection of urine over 24 h can be cumbersome, so a simpler sampling method would be more convenient for patients. Our results show that the AP(CaOx) and Ca/Cit in 12-h overnight urine samples were significantly different in stone-forming and healthy individuals. Moreover, the overnight samples had higher values for both risk indexes than the 12-h daytime samples and the 24-h samples. These findings support previous evidence that averaging results from the whole day masks peaks of lithogenic risk that occur at nighttime [14, 26]. Therefore, the analysis of a 12-h overnight sample is more convenient for patients and appears to be more sensitive in detection of increased risk of crystallization of urine. Considering that the night is a period of high urinary crystallization risk, the advice of increasing fluid intake at the late evening should be strongly encouraged in order to decrease urinary supersaturation degree.

Our data on the AP(CaP) index indicated the highest values were in daytime urine. This is due to diurnal variations in pH, which strongly affects calcium phosphate solubility [27, 28]. Urinary pH increases during the day, and so does calcium phosphate supersaturation [29]. However, we think that use of overnight urine is preferable to daytime or 24-h urine for evaluation of the risk of calcium phosphate stones. Remarkably, we found that the AP(CaP) index had a much wider range in daytime than in overnight urine, and also had more overlap in relation to stone type. The influence of punctual food intake on urinary pH can explain this daytime variability. At night, fasting makes pH values decrease and stabilize [30], so the differences between patients with pure calcium phosphate stones and other types of stones were more evident.

In agreement with other studies, we observed a correlation between the indexes and renal stone type in adults. Thus, we believe that these risk indexes might be helpful in cases when analysis of calculus composition is unavailable, because the results may suggest the chemical composition of the stone.

A limitation of this study is that we only enrolled a small number of stone-forming children because renal lithiasis is very rare at this age. However, our observation of similar patterns in children and adults suggest that the findings of the larger adult group may also be applicable to children. Another limitation is that we did not have the stone composition for all patients, and that very few patients had HAP stones. However, our results provide a foundation for further studies of renal lithiasis in children and of the relationship of different indexes with stone composition. Furthermore, more data is warranted, including a higher number of patients, in order to help determine a cutoff value for the studied indexes.

## Conclusions

Calculation of renal lithiasis risk indexes is easy and may facilitate the decision-making process for treatment of stone-forming patients because the indexes, which integrate the results of multiple urinary parameters, have shown higher values in stone forming patients that in healthy subjects. These differences have been more evident in the 12 h overnight urine sample. Our results indicate that use of a 12-h overnight urine sample should be considered as a complementary information to that provided by the analysis of 24-h samples when evaluating lithogenic risk, because collection of the overnight sample is more convenient and has higher values in all tested indexes.

## Abbreviations

AP(CaOx): ion activity product of calcium oxalate
AP(CaP): ion activity product of calcium phosphate
Ca: calcium
Cit: citrate
COD: calcium oxalate dihydrate
COMp: papillary calcium oxalate monohydrate
COMu: unattached calcium oxalate monohydrate
HAP: hydroxyapatite
